# Effect of Heat Treatment Conditions on Corrosion Resistance of 0.28C–1.4Mn–0.3Si–0.26Cr Steel with Nb, Ti, and V Microadditions

**DOI:** 10.3390/ma14123254

**Published:** 2021-06-12

**Authors:** Anna Wojtacha, Monika Kciuk, Marek Opiela

**Affiliations:** Department of Engineering Materials and Biomaterials, Silesian University of Technology, 18A Konarskiego Street, 44-100 Gliwice, Poland; monika.kciuk@polsl.pl (M.K.); Marek.Opiela@polsl.pl (M.O.)

**Keywords:** corrosion resistance, heat treatment, HSLA-type steel, microstructure

## Abstract

The article presents the results of the research on the influence of heat treatment conditions on corrosion resistance of newly developed HSLA-type (High Strength Low Alloy) steel in selected corrosive environments. Laboratory tests were carried out with using a salt spray chamber, enabling the continuous spraying of brine mist (5% NaCl) during 96 h under high humidity conditions. Additionally, as part of corrosion experiments, tests were carried out using the gravimetric method, in which the intensity of corrosive processes was measured by the linear corrosion rate. The research conducted revealed that the best corrosion resistance was noted for steel with a high-temperature tempered martensite microstructure. Investigated 0.28C–1.4Mn–0.3Si–0.26Cr steel with Nb, Ti, and V microadditions can be used in offshore drilling constructions and production platforms exposed to salts present in sea water, chlorides, sulfates, carbonates, and bromides, among others.

## 1. Introduction

The problem concerning the durability of metallic materials in natural and artificial environments is exceptionally important during both the design stage and operation of constructions and devices. Immense economic losses due to the corrosion of metal materials are the result of their chemical or electrochemical interaction with the surrounding chemically active environment. Corrosion damage results in continuous reduction of the effective cross-section of constructions, parts of machines and devices, and thus decreasing the strength and performance properties over the time of their operation, with a simultaneous increase in stress without the load change [1,2,3]. This creates the necessity to periodically replace corrosion-damaged parts, often subjected to other wear mechanisms, causing weakening of operational values, and ultimately leading to unexpected failures, often hazardous to the environment [4,5,6,7,8].

Modern constructional steels should be characterized not only by high strength, crack resistance, good weldability, and formability, but also low weight and guaranteed service life of the constructions made of these steels. The HSLA-type steels, usually containing up to about 0.2% C and about 1.5% Mn as well as microadditions with high chemical affinity for carbon and nitrogen (Nb, Ti, and V up to 0.1%), sometimes with increased concentration of N, and in the case of toughening steel also up to 0.005% B–increasing hardenability, broadly meet the mentioned requirements [9,10,11,12,13,14]. The HSLA-type steels are widely used in many industries due to their high strength-to-weight ratio and high ductility at low production costs. Both technical and economic aspects determine that HSLA-type microalloyed steels are widely used in the construction of highly loaded welded structures operated in various temperature-stress conditions (e.g., oil and gas pipelines, drilling platforms, bridges, means of heavy transport, self-propelled lifting and port devices [15,16,17]).

Very interesting research results were presented in the works of Khalaj et al. [18,19] in which artificial neural networks were used to monitor the corrosion of HSLA-type steels assigned to the construction of pipelines. Based on the experimental data, a model was developed in which the input data were, among others, the chemical composition of steel, microstructure, and corrosion cell characteristics. It was revealed that the elaborated model could be used to monitor the corrosion process of microalloyed steels in a wide range of their chemical composition. In [20], electrochemical tests of API-X70 steel were carried out in order to connect the microstructure characteristics of the heat-affected zone with their corrosion properties. Electrochemical tests were used to evaluate the kinetics of corrosion, dissolution, and passivation. The authors in [21] presented the research results of uniaxial cyclic creep and incremental damage of HSLA-type steel pipes under various conditions accelerating corrosion. The impact of exposure time and the size of the load on the cyclic and monotonic increase in the local load as well as the number of cycles causing corrosion damage were investigated. It was shown that the effects of corrosion on the examined steel were more distinct in the case of cyclic loading compared to monotonic loading, and the creep rate and the number of cycles significantly increased the susceptibility of the steel to corrosion. Similar issues concerning corrosion resistance of HSLA-type steels were the subject of research with the results presented in [22,23,24,25,26,27].

The mentioned works do not change the fact that most studies on HSLA-type steels have focused on the influence of the microadditions and thermo-mechanical treatment conditions on the mechanical properties. Therefore, there is a need to determine their service life when operating in a corroding medium, particularly for new steel grades.

The aim of this study was to investigate the effect of heat treatment conditions on the corrosion resistance of newly developed HSLA-type constructional steel with Nb, Ti, and V microadditions in the selected corroding mediums.

## 2. Material and Experiments

The tests were carried out on the newly elaborated microalloyed HSLA-type steel with the chemical composition listed in Table 1. The laboratory ingot of 100 kg was modified with rare earth elements (0.056% Ce, 0.030% La, and 0.022% Nd) to suppress the deformability of non-metallic inclusions. Casting was performed in an argon atmosphere. Initial plastic working of ingots into flat bars with a cross-section of 30 mm × 150 mm was carried out with the method of open die forging on a hydraulic press in a temperature range of 1200–900 °C.

In order to obtain a diversified microstructure, samples of the examined steel were subjected to various heat treatment operations (i.e., normalizing, quenching, and also quenching and high-temperature tempering). Heating and annealing treatments during normalizing and quenching were carried out in a HTCT 03/16 type NABERTHERM electric chamber furnace (Carbolite Gero, Neuhausen, Germany), and during tempering in the P500 type PROGRAMAT furnace (Ivoclar Vivadent AG, Schaan, Liechtenstein). Detailed parameters of the performed heat treatment are presented in Table 2.

The examined steel, after quenching at the temperature of 900 °C and subsequent tempering at 600 °C, revealed the following mechanical properties: R_p0,2_ ~939 MPa, UTS ~990 MPa, TEl ~16%, RA ~58%, and KV^−40^ ~80 J [14].

Corrosion resistance of the investigated microalloyed steel was evaluated using gravimetric and potentiodynamic methods. Additionally, accelerated corrosion tests in a salt spray chamber were carried out in accordance with EN ISO 9227:2012 [28].

For the purpose of gravimetric tests, 27 specimens with dimensions of 20 mm × 10 mm x 14 mm were prepared, thoroughly degreased with acetone and subsequently dried. Samples were weighed on an analytical balance with the accuracy of 0.0001 g and then placed in 3.5% NaCl solution, in 0.1 M NaOH solution, and in a 0.1 M H_2_SO_4_ solution. They were removed after 168 h, washed with water, and reweighed. The corrosion rate was assessed based on the calculation of mass loss (V_c_) for each of tested samples, according to the dependence [29,30,31]:

(1) $$V_{C}=\frac{\Delta m}{S\cdot t}$$

where ∆m is the difference in mass of the sample before and after corrosion test; g, S is the area of the sample, m^2^; and t is the time of the corrosion test, day.

Subsequently, the unit of the V_p_ average wear rate of the cross-section was calculated, which expresses the reduction in the transverse dimension of sample by 1 mm during a year. Average corrosion rate V_p_ is calculated from the average mass rate V_c_, according to the equation [29,30]:

(2) $$V_{p}=\frac{V_{c}\cdot 365}{1000\cdot \rho }$$

where ρ is the density, g/cm^3^.

**Figure 1:**
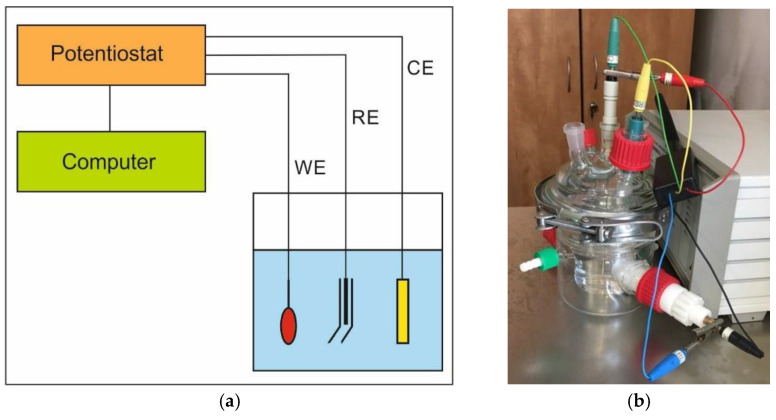
Measuring system scheme (**a**) and the set (**b**) used for the potentiodynamic tests.

Potentiodynamic tests were carried out using a Potentiostat-Galvanostat ATLAS 0531 EU device (ATLAS-SOLLICH, Gdańsk, Poland). Prior to testing, specimens with an average surface area of 100 mm^2^ were cleaned in ethyl alcohol. The sample of examined steel was the high-potential working electrode (WE), and Ag/AgCl electrode—the reference electrode (RE), immersed in a conductive solution in the Luggin’s capillary. The auxiliary electrode (CE) was made of stainless steel. The corroding medium was a 3.5% NaCl solution with a volume of 160 mL. The diagram of the implemented meter circuit is presented in Figure 1a, and its view in Figure 1b. The results of the performed tests were generated with the use of the AtlasCorr05 software (Rębiechowo, Poland).

Corrosion resistance of the investigated steel was additionally evaluated based on the salt spray chamber accelerated tests. First, samples were weighed using an analytical balance along with measuring their geometrical dimensions. Subsequently, specimens were placed in a chamber with a neutral salt mist atmosphere, obtained through continuous spraying of an aqueous solution of 5% sodium chloride for 96 h. The temperature in the chamber was constant throughout the experiment and was equal to 35 °C. With the aim to test corrosion resistance, several criteria were assumed including the appearance of samples after removing the surface corrosion products, mass loss, time to the first indications of corrosion, the type, and distribution of corrosion damage.

Microstructure observations were carried out using light and scanning microscopy. Preparation of specimens for metallographic tests included standard grinding and mechanical polishing procedures. Metallographic specimens were etched in Nital. Microstructure observations were conducted using a Z1m ZEISS Axio Observer light microscope (Carl Zeiss AG, Jena, Germany) in a magnification range from 100× to 500×. Fractographic studies were performed with a SUPRA 35 high-resolution scanning electron microscope (Carl Zeiss AG, Jena, Germany), applying an accelerating voltage of 20 kV and magnifying power ranging from 100× to 15,000×. Identification of the chemical composition of corrosion products on the surface of samples was carried out using an EDS energy dispersive x-ray spectrometer (EDAX TRIDENT XM4, Mahwah, NJ, USA).

## 3. Results

**Figure 2:**
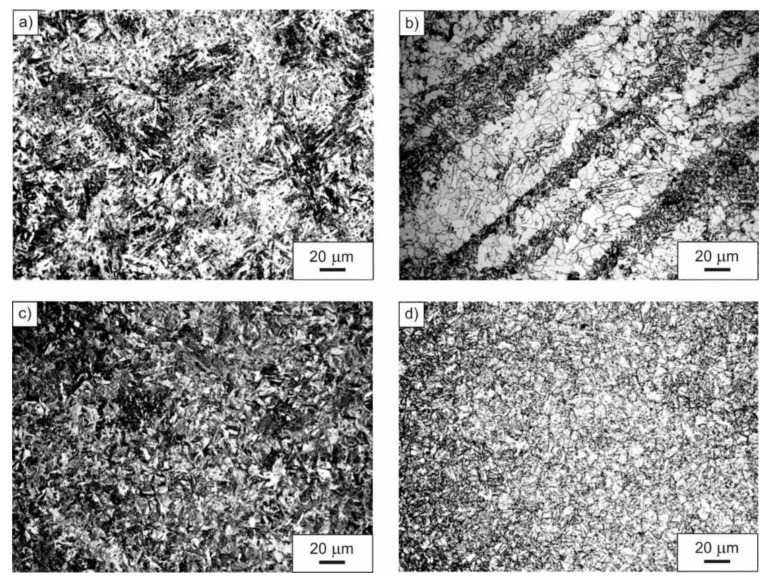
Microstructure of the examined microalloyed steel in as-delivered condition (**a**), after normalizing (**b**), after quenching (**c**), after quenching and high-temperature tempering (**d**).

Diversified microstructure of the examined microalloyed steel after applied heat treatment operations is shown in Figure 2. The steel in as-delivered condition revealed heterogeneous ferritic-pearlitic microstructure with a prevailing portion of the α phase in the acicular form (Figure 2a). Normalizing done at the temperature of 900 °C, with subsequent open air cooling, resulted in the formation of a fine-grained, band-like ferritic-pearlitic microstructure (Figure 2b). Fine lath martensite, obtained after austenitizing at the temperature of 900 °C for 20 min and subsequent quenching in water, is shown in Figure 2c, whereas Figure 2d presents a fine-grained microstructure of high-temperature tempered martensite with visible grain boundaries of prior austenite. The microstructure presented in Figure 2d was obtained as a result of quenching in water after austenitizing at the temperature of 900 °C and successive high-temperature tempering at the temperature of 600 °C.

Detailed results of the corrosion resistance tests of the examined steel determined with the use of the gravimetric method and expressed as the corrosion rate (i.e., decrease in the effective cross-section of sample in mm/year) are presented in Table 3. The dataset together in this table shows that the corrosion resistance of the investigated steel depends on both the structural condition and applied corroding medium. Performed tests revealed that the studied steel in the quenched and tempered condition with the microstructure of high-temperature tempered martensite was most corrosion resistant, regardless of the acidity of the affecting medium. The lowest average corrosion rate of 0.0076 mm/year in the mentioned structural state was observed during testing in a 0.1 M solution of NaOH. In this medium, the highest average corrosion rate of 0.2308 mm/year was noted in the case of steel after normalizing with a ferritic-pearlitic microstructure. Performed research pointed out that the examined steel was least corrosion resistant in 0.1 M solution of H_2_SO_4_. The average corrosion rates, obtained for this medium, were 1.9169 mm/year, 1.0445 mm/year, and 1.0083 mm/year for steel with a ferritic-pearlitic, martensitic, and high-temperature tempered martensite microstructure, respectively. The tests, performed with the use of the gravimetric method, revealed that the steel in the quenched and tempered condition (i.e., after quenching and high-temperature tempering) showed very good corrosion resistance in 0.1 M NaOH solution, corrosion resistance in 3.5% NaCl solution, and low corrosion resistance in 0.1 M H_2_SO_4_ solution. Steel with ferritic-pearlitic microstructure, obtained after normalizing, had the lowest corrosion resistance regardless of the type of the environment used.

**Figure 3:**
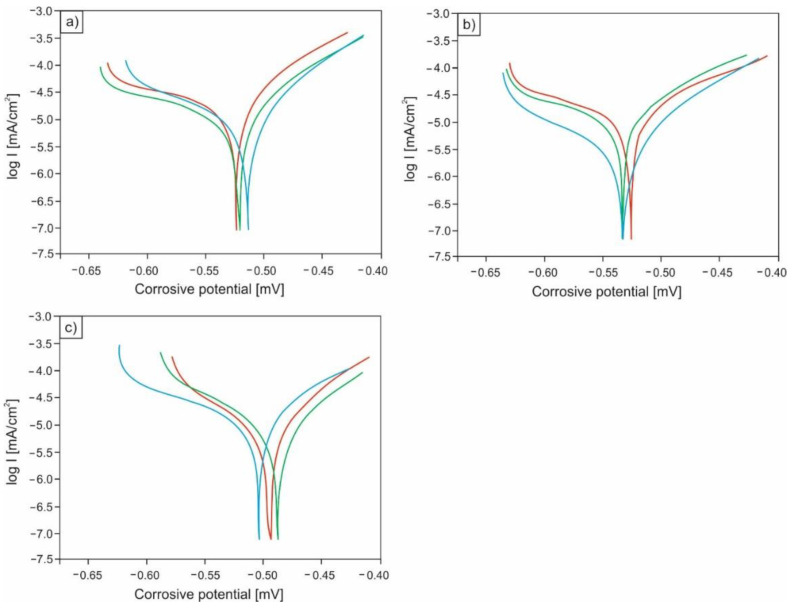
Changes in current density as a function of corrosion potential for specimens after normalizing (**a**), after quenching (**b**), and for samples after quenching and high-temperature tempering (**c**).

The conducted potentiodynamic tests showed that the corrosion resistance of the analyzed microalloyed steel noticeably depends on the microstructure, the differentiation of which resulted from various heat treatment operations. Based on the performed research, it can be concluded that the highest corrosion resistance is demonstrated by specimens in the quenched and tempered condition (after quenching and high-temperature tempering) with the microstructure of high-temperature tempered martensite. The average value of corrosion current for this structural state was equal to 0.008 mA/cm^2^, while the average polarization resistance was equal to 2.338 kΩ·cm^2^. Slightly higher average corrosion current (0.012 mA/cm^2^) was observed in the case of specimens in quenched condition with a martensitic microstructure. The lowest corrosion resistance was revealed for samples with a ferritic-pearlitic microstructure (after normalizing). For this structural state, a distinct increase in the average value of corrosion current to 0.023 mA/cm^2^ and a clear decrease in the mean value of polarization resistance to 1.447 kΩ·cm^2^ were demonstrated. Detailed results of the conducted potentiodynamic tests are set together in Table 4, while Figure 3 shows a comparison of the current density changes as a function of corrosion potential for two structural states: after normalizing (Figure 3a) and after quenching and high-temperature tempering (Figure 3b).

The results of the corrosion resistance tests done with the use of a salt chamber, enabling continuous spraying of brine mist (5% NaCl) for 96 h under high humidity conditions, are summarized in Table 5. The data presented in this table correlate with the test results of the impact of heat treatment on the corrosion resistance of the analyzed steel, obtained with the use of the gravimetric method. The performed research showed that the lowest average corrosion rate of 5.97 mm/year was recorded in the case of samples with a microstructure of high-temperature tempered martensite. A slightly higher average corrosion rate of approximately 6.38 mm/year was observed for specimens in quenched condition with a martensitic microstructure whereas specimens after normalizing with a two-phase ferritic-pearlitic microstructure were least corrosion resistant. For this structural state, the average corrosion rate was equal to 17.99 mm/year. Significantly higher average corrosion rate, obtained in the salt spray chamber, compared to the values obtained with the use of the gravimetric method, were the result of a higher concentration of Cl^−^ ions and a higher test temperature (35 °C).

**Figure 4:**
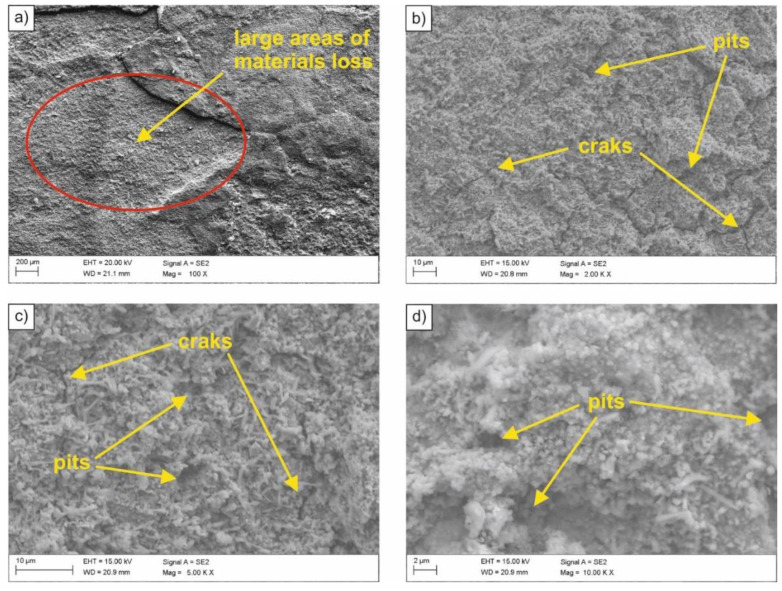
(**a**–**d**) SEM image of the surface of the sample subjected to normalizing after exposure in 0.1 M sulfuric acid (VI) solution.

In order to determine the type of corrosion and the nature of corrosion damage, fractographic tests were carried out using a scanning electron microscope. The surface of the sample with the lowest corrosion resistance (i.e., after normalizing, exposed to 0.1M solution of sulfuric acid (VI)) is shown in Figure 4. Based on the conducted observations, numerous material decrements were found in relatively large areas of the sample (Figure 4a). Moreover, cracks (Figure 4b) and numerous pits of various shapes (Figure 4c,d) were revealed on the sample surface. Observed corrosion effects were caused by lowering the pH and the material becoming active. As a result of direct contact with aggressive medium, the tested HSLA-type microalloyed steel underwent an accelerated process of corrosion, in which, in addition to oxygen depolarization, there was also depolarization with the use of hydrogen ions.

**Figure 5:**
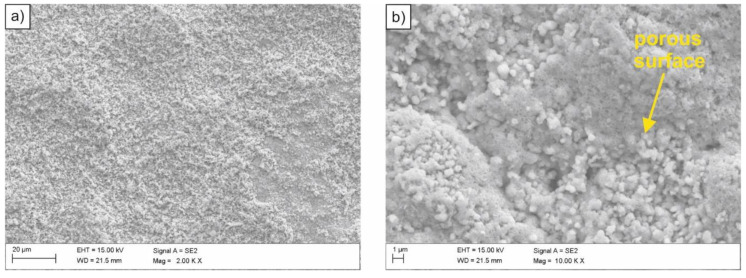
(**a**,**b**) SEM image of the surface of the sample subjected to normalizing after exposure in the 3.5% NaCl solution.

The examined steel samples, exposed to inactive medium (3.5% NaCl solution), underwent uniform corrosion over the entire surface of the material contact with corroding medium (Figure 5). This was caused by the action of corrosion micro-cells evenly distributed over the entire corroded surface. Similar to an acidic environment, oxygen reduction and hydrogen secretion are possible cathode processes. However, due to a too low concentration of hydrogen ions, their reduction practically did not take place. Oxygen depolarization occurred on the sample surface, and its rate depended on the access of oxygen to the material surface. After some time, corrosion products appear on the surface, which decreases the rate of corrosion processes. Therefore, the observed corrosion rate of the tested HSLA-type steel in inactive medium was lower compared to the corrosion rate in the acidic medium. Moreover, it was found that the sample surface was porous (Figure 5b).

**Figure 6:**
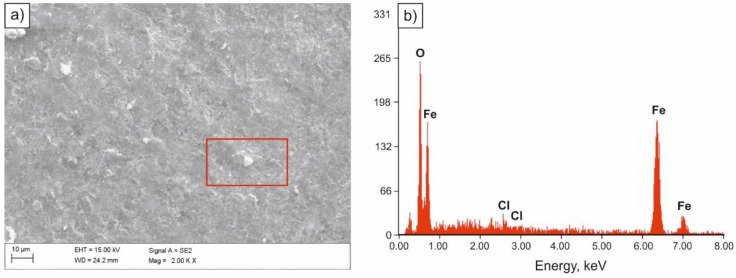
SEM image of the surface of the sample subjected to normalizing after tests in the salt spray chamber (**a**), spectrum of the corrosion product (**b**).

Specimens exposed to 5% NaCl solution in the salt spray chamber were subjected to pitting corrosion as a result of the aggressive impact of chloride ions. Corrosion attack occurred in areas with the smallest thickness of the protective layer, damage that resulted in uncovering small fragments of the steel substrate and transition of metal ions into the solution. As a result of their partial hydrolysis and the presence of H+ hydrogen ions, the inside of pitting became acidified, while the concentration of chloride ions increased and the concentration of oxygen decreased [32,33]. Pitting development is autocatalytic. Partially exfoliated surface of the sample in as-annealed condition after the salt spray chamber tests is presented in Figure 6a, and in Figure 6b, the spectrum of the chloride corrosion product.

**Figure 7:**
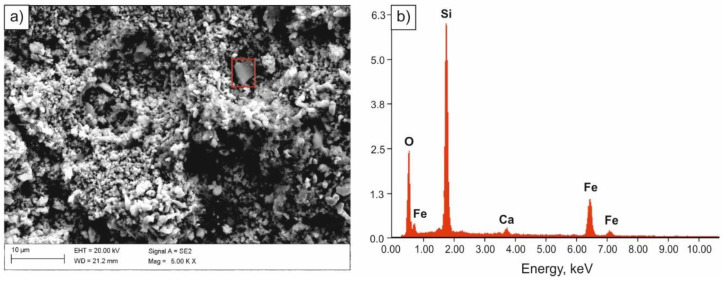
SEM image of the surface of the sample subjected to normalizing after tests in 0.1 M solution of NaOH (**a**), spectrum of the corrosion product (**b**).

**Figure 8:**
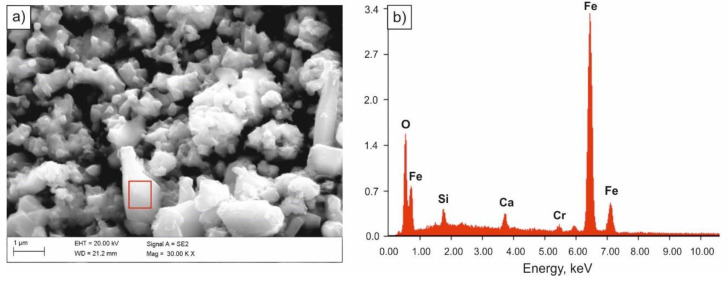
SEM image of the surface of the sample subjected to quenching after tests in 0.1 M solution of NaOH (**a**), spectrum of the corrosion product (**b**).

Specimens immersed in 0.1 M solution of NaOH were most corrosion resistant. Solid corrosion products, formed on their surfaces, probably slowed down the corrosion processes, almost completely limiting the anodic process. However, a diversified nature of the damage of the sample surface was found. Few pits with oxide corrosion products could be observed in the individual areas of the sample (Figure 7 and Figure 8).

## 4. Discussion

The corrosion cells in metals are formed as a result of defects or mechanical or chemical damage to the passive layer. The presence of a cathodic process supporting factor, most often in a form of dissolved oxygen, is necessary in a neutral solution for the corrosion process to occur. The active-passive cell, which determines the local corrosion process of metals, is a type of concentration cell with different aeration. Metals are most often subjected to pitting corrosion, which takes the form of oval pits. Corrosion is initiated in the areas where the weakening of the oxide layer has occurred. A necessary condition for pitting corrosion to take place is exceeding the minimum potential, called the breakthrough potential in the area of metal passivity [33]. In the lowest thickness areas of the passive layer, there was a high decrease in the potential, which accelerated the penetration of Cl^−^ ions. After small areas of the substrate metal are locally uncovered, metal ions transit into the solution. Partial hydrolysis takes place along with the formation of basic salts and H+ ions, acidifying the pitting microenvironment. Metal ions, migrating from the pitting area to the solution under the impact of Cl^−^, precipitate in the form of insoluble hydroxides on the pitting surface [34,35].

On the basis of performed gravimetric tests, the influence of the acidity of the medium (pH) on corrosion was revealed. The lowest corrosion rate was observed in NaOH solutions with alkaline pH, and the highest corrosion rate in H_2_SO_4_ acid solutions. In strong acid environments, the oxide layer, being a diffusion barrier located on the iron surface, dissolves at pH values below 4. In weaker acids, the oxide layer dissolves at higher pH values, hence the corrosion rate of iron, accompanied by hydrogen precipitation, increases already at pH equal to 5 or 6. In the case of a weaker acid, the quantity of H+ ions that can react and solubilize oxide layer is greater compared to the strong acid. Moreover, easier access of oxygen to the metal surface with dissolved layer of oxides promotes oxygen depolarization, which has the most considerable influence on the corrosion rate [5,7].

One of the basic medium parameters, affecting the degree of resistance to pitting corrosion, is the concentration of chloride ions. In the case of the NaCl solution, there was a higher conductivity because additional cathodes and anodes are formed at a much greater distance from each other. The reaction of NaOH with anodic FeCl_2_ at the cathodes did not take place immediately. Both substances diffuse into the solution and the interaction takes place to form Fe(OH)_2_. Iron hydroxide (II) does not form any protection on the metal surface, hence iron corrodes rapidly in NaCl solutions as more dissolved oxygen diffuses to the cathode areas. Decreasing solubility of oxygen becomes important at the concentration of NaCl above 3%, hence the decrease in the corrosion rate. Minimum concentration of Cl^−^ ions, leading to pitting corrosion, varies within very wide limits, depending on other parameters (i.e., environment, type of material, maximum concentration limit of these ions). Additionally, the concentration of Cl^−^ ions affects pitting corrosion incubation time; the higher the concentration and the higher the critical potential of pitting nucleation, the shorter the incubation time [5,34,35].

Corrosion resistance is strictly related to material microstructure, obtained as a result of the performed heat treatment. It can be concluded, based on the tests carried out, that after quenching and high-temperature tempering at 600 °C, the specimens were characterized by the lowest value of the average wear rate of cross-section V_p_ (i.e., the highest corrosion resistance). On the other hand, the lowest corrosion resistance was observed for samples subjected to normalizing at the temperature of 900 °C with ferritic-pearlitic microstructure. Pearlite is the eutectoid mixture, consisting of alternating ferrite and cementite plates, each of which reacts differently to the corroding medium. The anodic nature of ferrite in relation to cementite leads to the intensification of corrosion processes due to the formation of corrosion cells at the ferrite and cementite contact. Single-phase microstructure steels are more resistant to corrosion because, unlike in the case of multi-phase microstructure, the formation of local cells is less probable. Ferritic steels are characterized by a very good corrosion resistance due to the uniform distribution of ferrite. While cathode and anode regions are close to each other, there is no possibility of forming galvanic cells [36,37,38]. Steels with a strength higher than ferritic steels, with martensitic or bainitic microstructure, have lower corrosion resistance due to the presence of high density lattice defects that favor corrosion.

As already mentioned, the corrosion rate is an important parameter for estimating corrosion losses. Table 6 presents a six-point scale for the corrosion resistance of metals and metal alloys. Table 7 and Table 8 present the degree of corrosion resistance of the examined microalloyed steel specimens after various heat treatment operations, determined with the use of the gravimetric method and on the basis of the tests in the salt spray chamber.

Based on the data presented in Table 7 and Table 8, it can be concluded that the best corrosion resistance was demonstrated by the specimens after quenching and high-temperature tempering, with a microstructure of high-temperature tempered martensite. According to the classification of environments for temperate climate included in ISO 12944-2:2018 [40], steel with high corrosion resistance can be used even in industrial and coastal areas with medium salinity as well as in chemical plants or for elements used for shipbuilding.

Similar issues concerning the impact of the microstructure of high strength low alloy steels on corrosion resistance in various environments were the subject of research in [36,41,42,43,44,45]. Gou et al. [41] examined the effect of the behavior of diversified microstructure in 3.5% NaCl solution medium on the corrosion resistance of HSLA-type steels. The results of the mass loss measurements, obtained in this work, and the results of the potentiodynamic tests revealed that the fine-grained single-phase microstructure steel was characterized by the best corrosion resistance. Chen and Zhang, on the basis of tests of 0.12C–1.7Mn–0.25Si–0.6Cr steel with 0.06% Nb microaddition, revealed that corrosion resistance significantly depends on the microstructure and grain size, controlled by heat treatment and the precipitation of niobium carbides and niobium carbonitrides [42]. Sherif and Seikh [43] investigated the behavior of 0.12C–1.0Mn–0.1Si–0.07Al–0.060Nb steel in 1 N solution of H_2_SO_4_ after single and multiple quenching. This work showed that the corrosion rate depends on the obtained microstructure, which is a function of the applied heat treatment. The results presented in the above-mentioned studies fully correlate with the results of the analyzed HSLA-type microalloyed steel.

## 5. Conclusions

Corrosion resistance of the examined HSLA-type microalloyed steel significantly depends on the microstructure, which is a derivative of the applied heat treatment operations. Conducted comprehensive evaluation of corrosion resistance of 0.28C–1.40Mn–0.3Si–0.26Cr steel with Nb, Ti, and V microadditions with concentrations of 0.027%, 0.028%, and 0.019%, respectively, revealed that the least corrosion resistant were specimens after normalizing. In this condition, the steel shows a two-phase ferritic-pearlitic microstructure. Ferrite and cementite, forming a lamellar pearlite morphology, react differently to the presence of the corroding medium. The anodic nature of ferrite, in relation to cementite, leads to the intensification of corrosion processes due to the formation of corrosion cells at the contact of these two phases.

The research conducted with the use of the gravimetric method, potentiodynamic tests, and accelerated corrosion tests in the salt spray chamber revealed that the best corrosion resistance was noted for steel with aa high-temperature tempered martensite microstructure, obtained as a result of combined quenching and high-temperature tempering operations.

Moreover, on the basis of the performed research, the influence of the acidity of the medium on corrosion was revealed. The highest corrosion resistance of the examined microalloyed steel was observed in NaOH solutions with alkaline pH, and the lowest in H_2_SO_4_ acid solutions.

The investigated 0.28C–1.40Mn–0.3Si–0.26Cr steel with Nb, Ti, and V microadditions was corrosion resistant in the environment of chloride ions, hence it is possible to use it in the shipbuilding industry for vessel hulls. Due to its corrosion resistance in an artificial sea water environment (3.5% NaCl solution), the analyzed steel can also be used in offshore drilling constructions and production platforms that are exposed to salts present in sea water, chlorides, sulfates, carbonates, and bromides, among others.

## Figures and Tables

**Table 1:** Chemical composition of the investigated steel.

C	Mn	P	S	Si	Cr	Nb	Ti	V	B
0.28	1.40	0.008	0.004	0.30	0.26	0.027	0.028	0.019	0.003

**Table 2:** Parameters of the performed heat treatment of the investigated steel samples.

Heat Treatment Operation	Soaking Temperature, °C	Soaking Time, min	Cooling Medium
Normalizing annealing	900	20	Air
Quenching	900	20	Water
Tempering	600	60	Water

**Table 3:** Results of the gravimetric investigations.

Normalizing		Quenching		Quenching and High-Temperature Tempering	
Environment	V_p_ [mm/Year]	Environment	V_p_ [mm/Year]	Environment	V_p_ [mm/Year]
3.5% NaCl	0.2721	3.5% NaCl	0.0922	3.5% NaCl	0.0877
0.1M NaOH	0.2308	0.1M NaOH	0.0089	0.1M NaOH	0.0076
0.1M H_2_SO_4_	1.9169	0.1M H_2_SO_4_	1.0445	0.1M H_2_SO_4_	1.0083

**Table 4:** Results of the potentiodynamic investigations.

Normalizing		Quenching		Quenching and High-Temperature Tempering	
Corrosion Current, mA/cm^2^	0.023	Corrosion Current, mA/cm^2^	0.012	Corrosion Current, mA/cm^2^	0.008
Corrosive potential, mV	−523.19	Corrosive potential, mV	−543.50	Corrosive potential, mV	−505.96
polarization resistance, kΩ cm^2^	1.447	polarization resistance, kΩ cm^2^	1.731	polarization resistance, kΩ cm^2^	2.338

**Table 5:** Research results of the corrosion resistance tests in a salt spray chamber.

Normalizing	Quenching	Quenching and High-Temperature Tempering
V_p_ [mm/Year]	V_p_ [mm/Year]	V_p_ [mm/Year]
17.99	6.38	5.97

**Table 6:** Summary of corrosion resistance of metals and their alloys [39].

Corrosion Resistance Group		Degree of Corrosion Resistance	Corrosion Rate, V_p_ [mm/year]	Corrosion Durability, Tr * [year/mm]
Definition	Designation			
completely resistance	I	1	>0.001	does not specify
very resistant	II	2	0.001–0.005	does not specify
		3	0.005–0.01	
resistant	III	4	0.01–0.05	10–100
		5	0.05–0.1	
about less resistant	IV	6	0.1–0.5	–110
		7	0.5–1.0	
not very resistant	V	8	1.0–5.0	0.1–1.0
		9	5.0–10.0	
not resistant	VI	10	<10.0	>0.1

* Tr is the ratio of the corrosive environment operating time to the decrement in sample cross-section.

**Table 7:** Definitions of corrosion resistance and the degrees of corrosion resistance for average V_p_ results, determined with the use of the gravimetric method in various corroding mediums.

Environment	Normalizing		Quenching		Quenching and High-Temperature Tempering	
	Degree of Corrosion Resistance	Definitions of Corrosion Resistance	Degree of Corrosion Resistance	Definitions of Corrosion Resistance	Degree of Corrosion Resistance	Definitions of Corrosion Resistance
3.5% NaCl	6	about less resistant	5	resistant	5	resistant
0.1 M NaOH	6	about less resistant	3	very resistant	3	very resistant
0.1 M H_2_SO_4_	8	not very resistant	8	not very resistant	8	not very resistant

**Table 8:** Definitions of corrosion resistance and the degrees of corrosion resistance for average V_p_ results, determined on the basis of the tests in the salt spray chamber.

Environment	Normalizing		Quenching		Quenching and High-Temperature Tempering	
	Degree of Corrosion Resistance	Definitions of Corrosion Resistance	Degree of Corrosion Resistance	Definitions of Corrosion Resistance	Degree of Corrosion Resistance	Definitions of Corrosion Resistance
5% NaCl	10	not resistant	9	not very resistant	9	not very resistant

## Data Availability

Data sharing is not applicable to this article.

## References

[1] Bhagavathi L.R., Chaudhari G.P., Nath S.K. (2011). Mechanical and corrosion behavior of plain low carbon dual-phase steels. Mater. Des..

[2] Hou Y., Lei D., Li S., Yang W. (2016). Experimental investigation on corrosion effect on mechanical properties of buried metal pipes. Int. J. Corros..

[3] Handoko W., Pahlevani F., Sahajwalla V. (2018). Enhanning corrosion resistance and hardness properties of carbon steel through modification of microstructure. Materials.

[4] Dean S.W., Grab G.D. (1985). Corrosion of carbon steel by concentrated sulfuric acid. Mater. Perform..

[5] Grajcar A., Płachcińska A., Topolska S., Kciuk M. (2015). Effect of thermomechanical treatment on the corrosion behavior of Si- and Al-containing high-Mn austenitic steel with Nb and Ti micro-additions. Mater. Tehnol..

[6] Svamy Nadh V., Vasugi K. (2014). Corossion resistance for different types of steel under, alkaline solution. Int. Adv. Res. J. Sci. Eng. Technol..

[7] Bastidas D.M. (2020). Corrosion and protection of metals. Metals.

[8] Teng F., Guan Y.T., Zhu W.P. (2008). Effect of biofilm on cast iron pipe corrosion in drinking water distribution system: Corrosion scales characterization and microbial community structure investigation. Corros. Sci..

[9] Liu Y., Shi L., Liu C., Yu L. (2016). Effect of step quenching on microstructure and mechanical properties of HSLA steel. Mater. Sci. Eng. A.

[10] Illescas S., Fernández J., Guilemany J.M. (2008). Kinetics analysis of the austenitic grain growth in HSLA steel with a low carbon content. Mater. Lett..

[11] Opiela M., Grajcar A. (2018). Microstructure and anisotrophy of plastic properties of thermomechanically-processed HSLA-type steel plates. Metals.

[12] Grajcar A., Radwański K. (2014). Microstructural comparison of the thermomechanically treated and cold deformed Nb-microalloyed TRIP steel. Mater. Tehnol..

[13] Park D., Huh M., Shim J., Jung W. (2013). Strengthening mechanism of hot rolled Ti and Nb microalloyed HSLA steels containing Mo and W with various coiling temperature. Mater. Sci. Eng. A.

[14] Opiela M. (2014). Thermomechanical treatment of Ti-Nb-V-B microalloyed steel forgings. Mater. Tehnol..

[15] Show B.K., Veerababu R., Balamuralikrishnan R., Malokondaiah G. (2010). Effect of vanadium and titanium modification on the microstructure and mechanical properties of a microalloyed HSLA steel. Mater. Sci. Eng. A.

[16] Fernández J., Illescas S., Guilemany J.M. (2007). Effect of microalloying elements on the austenitic grain growth in a low carbon HSLA steel. Mater. Lett..

[17] Adamczyk J., Opiela M. (2004). Influence of the thermo-mechanical treatment parameters on the inhomogeneity of the austenite structure and mechanical properties of the Cr-Mo steel with Nb, Ti, and B microadditions. J. Mater. Process. Technol..

[18] Khalaj G., Pouraliakbar H., Arab N., Nazefakhari M. (2015). Correlation of passivation current density and potential by using chemical composition and corrosion cel characteristics in HSLA steel. Measurement.

[19] Narmania N., Zarei B., Pouraliakbar H., Khalaj G. (2015). Predictions of corrosion current density and potential by using chemical composition and corrosion cel characteristics in microalloyed pipeline steels. Measurement.

[20] Khalaj G., Khalaj M.-J. (2016). Investigating the corrosion of the Heat-Affected Zones (HAZs) of API-X70 pipline steels in aerated carbonate solution by electrochemical methods. Int. J. Press. Vessel. Pip..

[21] Talebi M., Zeinoddini M., Mo’tamedi M., Zandi A.P. (2018). Collapse of HSLA steel pipes under corrosion exposure and uniaxial ineelastic cycling. J. Constr. Steel Res..

[22] Gonzalez-Rodrigez J.G., Casales M., Salinas-Bravo V.M., Albarran J.L., Martinez L. (2002). Effect of microstructure on the stress corrosion cracking of X-80 pipeline steel in diluted sodium bicarbonate solutions. Corrosion.

[23] Qiao Q., Lu L., Fan E., Zhao J., Liu Y., Peng G., Huang Y., Li X. (2019). Effects of Nb on stress corrosion cracking of high-strength low-alloy steel in simulated seawater. Int. J. Hydrogen Energy.

[24] Mohammadi F., Eliyan F.F., Alfantazi A. (2012). Corrosion of simulated weld HAZ of API X-80 pipeline steel. Corros. Sci..

[25] Eliyan F.F., Alfantazi A. (2013). Corrosion of the Heat-Affected Zones (HAZs) of API X-100 pipeline steel in dilute bicarbonate solutions at 90 °C—An electrochemical evaluation. Corros. Sci..

[26] Eliyan F.F., Mahdi E.S., Alfantazi A. (2012). Elecrochemical evaluation of the corrosion behaviour of API X-100 pipeline steel in aerated bicarbonate solutions. Corros. Sci..

[27] Wu W., Wang Q., Yang L., Liu Z., Li X., Li Y. (2020). Corrosion and SCC initiation behawior of low-alloy high-strength steels microalloyed with Nb and Sb in a simulated polluted marine atmosphere. J. Mater. Res. Technol..

[28] EN ISO 9227:2012 (2021). Corrosion Tests in Artificial Atmospheres—Salt Spray Tests.

[29] Glinicka A., Imiełowski S., Ajdukiewicz C. (2015). Influence of uniformly distributed corrosion on the compressive capacity of selected thin—walled metal columns. Procedia Eng..

[30] Paik J.K., Lee J.M., Park J.H., Hwang J.S., Kim C.W. (2003). Time—Variant ultimate longitudinal strength of corroded bulk carriers. Mar. Struct..

[31] Fakuda M., Fujii K., Nakayama T., Matsui S. (2011). An evaluation method for the remaining strength of plate girder with local corrosion under sleepers. Procedia Eng..

[32] Jakubowski M. (2014). Influence of pitting corrosion on fatigue and corrosion fatigue of ship structures Part I. Pitting corrosion of ship structures. Pol. Marit. Res..

[33] Kciuk M., Lasok S. (2017). Corrosion resistance of X5CrNi18-10 stainless steel. Arch. Civ. Mech. Eng..

[34] Lee H.H., Uhlig H.H. (1972). Corrosion fatigue of type 4140 high strength steel. Matallurgical Mater. Trans. B.

[35] Uhlig H.H., King F.K. (1972). The Flade potential of iron passivated by various inorganic corrosion inhibitors. Matallurgical Mater. Trans. B.

[36] Qaban A., Naher S. (2019). Investigation of corrosion resistance of high-strength low-alloy (HSLA) steel in fresh and salt water for pipeline application. AIP Conference Proceedings.

[37] Qaban A., Naher S. (2018). Influence of Al content on the corrosion resistance of micro-alloyed hot rolled steel as a function of grain size. AIP Conference Proceedings.

[38] Sarin P., Snoeyink V.L., Lytle D.A., Kriven W.M. (2004). Iron corrosion scales: Model for scale growth, iron release and colored water formation. J. Environ. Eng..

[39] PN-H-04608:1978 Metal Corrosion—The Scale of Resistance of Metals to Corrosion.

[40] EN ISO 12944-2:2018 (2017). Paints and Varnishes—Corrosion Protection of Steel Structures by Protective Paint Systems—Part 2: Classification of Environments.

[41] Guo Y.B., Li C., Liu Y.C., Yu L.M., Ma Z.Q., Liu C.X. (2015). Effect of microstructure variation on the corrosion behawior of high-strength low-alloy steel in 3.5 wt% NaCl solution. Int. J. Miner. Metall. Mater..

[42] Chen Y.T., Zhang K.G. (2012). Influence of grain size on corrosion resistance of a HSLA steel. Adv. Mater. Res..

[43] Sherif E.S., Seikh A.H. (2012). Effect of grain refirement on the corrosion behaviour of microalloyed steel in sulphuric acid solutions. Int. J. Electrochem. Sci..

[44] Ralston K.D., Birbilis N., Davies C.H. (2010). Revealing the relationship between grain size and corrosion rate of metals. Scr. Mater..

[45] Hadzima B., Janeček M., Estrin Y., Kim H.S. (2007). Microstructure and corrosion properties of ultra-grained interstitial free steel. Mater. Sci. Eng. A.

